# Lingering symptoms in non-hospitalized patients with COVID-19 – a prospective survey study of symptom expression and effects on mental health in Germany

**DOI:** 10.1186/s12875-025-02784-3

**Published:** 2025-04-02

**Authors:** Jörn Rohde, René Bundschuh, Yvonne Kaußner, Anne Simmenroth

**Affiliations:** 1 Department of General Practice, University Hospital Wuerzburg, Würzburg, Germany; 2 Counseling Center for Employees, University Hospital Wuerzburg, Würzburg, Germany

**Keywords:** Post-COVID, Long-COVID, Primary care, Mental health

## Abstract

**Background:**

The infection rates with SARS-CoV 2 virus, known since 2019, are currently significantly weakened in their dynamics. Nevertheless, COVID 19 is still a common disease, which in most cases is overcome quite well and can be treated by the general practitioner. Despite an initially uncomplicated disease progression, the long-term consequences can be considerable. Symptoms persisting over a period of more than 12 weeks after infection are summarized as Post-COVID (PC) syndrome. The aim of this study is to document the symptom expression in PC patients in the outpatient setting, with a major focus on limitations in daily life and consequences for mental health.

**Methods:**

This survey is part of a prospective European collaborative study with the German cohort having been slightly extended and evaluated separately. Data collection was performed by telephone interviews of adult SARS CoV 2 positive patients using standardized questionnaires (38 open and 6 closed questions). After an inclusion interview, follow-up interviews were conducted every 4 weeks over a period of 6 months. Participants were recruited in collaboration with the local health department (Wuerzburg, Germany).

**Results:**

Sixty participants were recruited in April and May 2021. After 12 weeks (PC cutoff), 48.3% still reported symptoms related to SARS-CoV-2 infection. The most commonly reported symptoms were fatigue/tiredness (33.3%), reduced concentration (26.7%), and shortness of breath (23.3%). One-quarter of respondents reported impaired functioning, with the most common daily limitations being sports (28.3%), work (25.0%), and social life (15.0%). At 6 months, 21.6% of respondents experienced anxiety and 11.6% reported depressive symptoms. Overall, 40.0% of respondents were concerned that their health would deteriorate again or not fully normalize because of COVID-19. Over two-thirds (70.0%) visited a physician during the course of the study because of COVID-19, 73.8% of whom visited their general practitioner.

**Conclusion:**

PC in outpatient care appears to be a complex and multifaceted condition that not only presents with physical symptoms, but also has a significant impact on mental health and daily life. Although the complexity of the condition is not yet fully understood, our findings suggest that it presents long-term challenges, particularly in outpatient care. Further research, particularly in larger and more diverse cohorts, is needed to confirm these observations. Routine screening for psychosocial comorbidities could be a valuable approach to identify supportive interventions that may help to reduce the risk of chronification and/or somatization.

**Supplementary Information:**

The online version contains supplementary material available at 10.1186/s12875-025-02784-3.

## Introduction

The SARS-CoV-2 (severe acute respiratory syndrome coronavirus type 2) was first mentioned in 2019 [1]. In the following years, there were several waves of infection with rapidly increasing case numbers worldwide [2]. Currently, the dynamics have been mitigated, but there are still new infections every day [2, 3]. Even though most people have become accustomed to deal with SARS-CoV-2, an infection can be a life-changing event for individual patients.

To date, more than 777 million (March 2025) COVID-19 cases have been recorded worldwide [2]. Mostly, the disease is well survived and can be managed in primary care settings. In approximately 4.6/100,000 of the cases, a hospital admission becomes necessary [4]. Even if the initial illness does not turn into a life-threatening event, the long-term consequences after an acute infection can be significant [5].

The WHO (World Health Organization) defines Post-COVID (PC) as “a history of probable or confirmed SARS-CoV-2 infection, usually 3 months from the onset of COVID-19 with symptoms that last for at least 2 months and cannot be explained by an alternative diagnosis.” [6]. However, a precise syndrome description for long-term sequelae after COVID-19 is not yet available. The observed symptoms are very diverse and not yet classified with certainty in their relevance [7, 8]. Various biological mechanisms that lead to PC are being discussed, but a conclusive pathophysiological explanation has yet to be found [8]. Certainly, mental and cognitive symptoms are particularly distressing for those affected. Meta-analyses have shown that 35% of people suffering from COVID-19 develop depressive symptoms [9]. Furthermore, around a third suffer from persistent fatigue, a quarter complains of dyspnoe or sleep disorder and a fifth show cognitive impairment [10, 11]. Valuable studies from Germany, with a comparable recruitment period and outpatient target group, identified persistent symptoms lasting at least 12 weeks in 34% to 46% of subjects [12, 13]. In comparison to the retrospective data collection used there with only a single application of the questionnaires, we offer a valuable addition with a prospective concept and multiple, close follow-up over time. Furthermore, we placed additional emphasis on the severity of symptoms and a differentiated consideration of mental problems. Our method enabled us to generate a gapless dataset of a cohort from the beginning of the pandemic. This not only holds historical significance but also serves as a valuable reference for future studies.

This present study was designed based on the CALIP study (COVID-19 and lingering symptoms in primary care patients), which, to our knowledge, was back then in 2021 the first prospective study in Europe to provide structured follow-up after SARS-CoV-2 infections in the outpatient care. The CALIP study recruited COVID-19 patients in eleven European countries and has recently been published [14]. For this study, we performed the recruitment and follow-up protocol in Germany and decided, out of our own interest, to increase the number of subjects, extend the existing protocol (Supplement 1) in order to analyze it separately for Germany.

This study investigates the long-term physical and psychosocial impacts of Post-COVID in non-hospitalized patients. In contrast to the predominantly present literature our study offers a purely outpatient and largely unvaccinated collective and extends existing research while exploring not only the purely physical symptoms but also the overall effects on everyday life as well as further mental symptoms.

## Material and Methods

### Study design

This study is based on a longitudinal, prospective design developed as an extension of the European CALIP study limited to the area of Wuerzburg, Bavaria [14]. The initial CALIP study required structured follow-up through telephone interviews (assessment of symptoms, impact of symptoms on daily activities and visits to general practice) these were conducted at weeks 2, 4, 8 and 12 [14]. Telephone follow-up has already proven to be practical and useful in other studies on the course of Covid-19 [15]. We expanded the protocol to include questions on psychosocial issues and economic topics. Furthermore, we added an additional final interview after six months to reassess persisting symptoms and set a focus on mental symptoms. The increase in the number of participants and the expansion of the subject areas enabled us to achieve independent and new results that do not compete with the CALIP Study in terms of content, nor are there major overlaps in the results or conclusions.

Target population were non-hospitalized adults with laboratory- confirmed SARS-CoV-2 infection and symptoms compatible with COVID-19. A maximum of two weeks between positive SARS-CoV-2 PCR and study inclusion was required. Data collection was solely based on telephone interviews using standardized questionnaires as described below.

### Study population and patient recruitment

The recruitment of participants was conducted from April 2021 to May 2021. During this time, the COVID-19 pandemic was in the rise of the third wave in Germany, which was largely dominated by the Alpha variant (B.1.1.7) [2, 16, 17]. If a person tested positive for SARS-CoV-2 in Wuerzburg and surrounding, an official notification was sent to the local health department. From there, all individuals received an information letter about the study and an invitation to participate. The patients independently contacted the Department of General Practice at the University Hospital of Wuerzburg. As inclusion criteria, we defined a laboratory-confirmed positive SARS-CoV-2 test, symptoms compatible with COVID-19 and the age of majority. Exclusion occurred if the subject was hospitalized due to COVID-19. Enrollment in the study took place after a physician had explained the study to the patient by telephone and the patient had signed a consent form, which was sent by mail/post. Withdrawal from the study was possible at any time.

### Data management/collection

For pseudonymization of the study participants, each person received a study identification number. After completion of the data collection, the contact details and names of the study participants were permanently deleted to provide an anonymized data set.

**Fig. 1:**
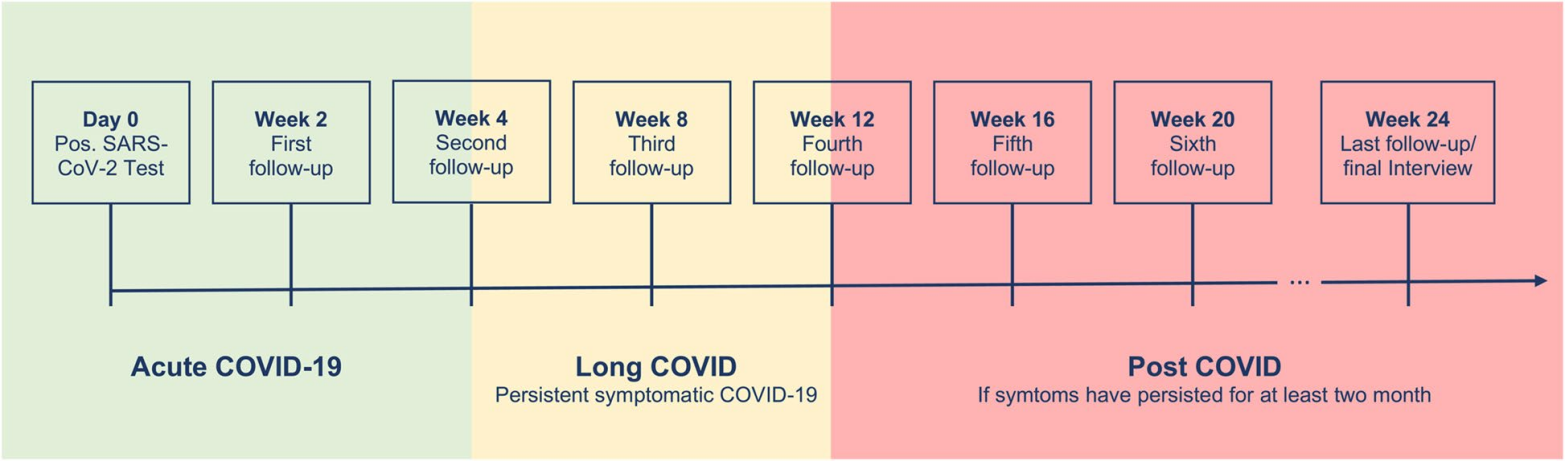
Timeline of systematic follow-up dates and definition for Long/Post-COVID. Interviews were generally conducted every four weeks. An additional interview took place two weeks after study inclusion. Green area: Acute infection; Yellow area: Long COVID if symptoms persisted after week four (Long-COVID threshold); Red area: Post-COVID if symptoms persisted for at least two months

Each study participant was interviewed at predetermined time intervals (Fig. 1). The duration of the follow-up was generally a minimum of 12 weeks and a maximum of six months. After week 12, further data was only collected until complete lack of symptoms was reported on two consecutive interviews. In addition, a final interview was held with all recruited study participants after six months. All interviews were conducted by the same person (RB, doctoral student).

The Ethics Committee of the University Hospital of Würzburg approved the procedure (file number: 4/21-me).

### Survey and statistical analysis

The central aim of the questionnaires was to assess the occurrence of various typical COVID-19 symptoms (including respiratory, gastrointestinal, neurological manifestations) and their intensity (very mild, mild, moderate, severe, very severe). In addition, the effects (none, light, moderate, quite strong, extreme) on daily activities (e.g. work, education, sports, social life) or the need for medical consultations or therapeutic measures were recorded.

Another central point was the assessment of psychological effects. In each interview, we asked about general mental symptoms, which could be restlessness, anxiety or depressive mood. The symptoms were summarized in one item. If one or more mental symptoms were present, subjects rated the intensity on a Likert scale (very mild, mild, moderate, severe, very severe). In the final interview, we separately asked about anxiety, depressive mood and concerns about the prognosis of COVID-19-specific symptoms. Complementary, the GAD-7 questionnaire (Generalized anxiety disorder 7) was used as a reliable tool at the final interview [18]. Furthermore, sociodemographic data (e.g., height, age, weight, smoking status, vaccination status, preexisting conditions, long-term medication) were collected at study inclusion (Table 1). Duration of work disability due to symptoms of COVID-19 disease was asked by offering five response categories (< = 5; 6–10; 11–15; 16–20; > 20 days) at the final interview. Before the questionnaire was published, it was piloted several times by specialists (doctors, psychologists) and affected persons (Sars-CoV 2 positive) to detect possible errors in consistency, response tendencies and desired behavior. Data were stored on a password-protected server in the Department of General Practice to which only two persons (RB, JR) had access.

**Table 1:** Clinical and sociodemographic characteristics of the study population

Characteristics	*n*		(%)	
Total	60		(100)	
Male	24		(40)	
Female	36		(60)	
	mean (sd)		md (min.-max.)	
Age	45.4	(14.9)	43	(21–76)
Height	1.73	(0.1)	1.73	(1.52–1.95)
Weight	74	(15.9)	70	(52–117)
BMI	24.5	(3.8)	24.1	(17.1–33.7)
	n		(%)	
Current Smoker	6		(10)	
Ex Smoker	18		(30)	
Employed	53		(88)	
Medical task	9		(15)	
Social task	13		(21.7)	
Office work	21		(35)	
Artisanal/industrial task	4		(6.7)	
Students	5		(8.3)	
Others	1		(1.7)	
Comorbidity	38		(63.3)	
Chronic respiratory disease	4		(6.7)	
Diabetes mellitus	3		(5)	
Cardiovascular disease	15		(25)	
Autoimmune disease	4		(5.1)	
Neurological disease	1		(1.7)	
Tumor disease	4		(6.7)	
Mental disease	4		(6.7)	
Others	16		(26.7)	
First SARS-CoV-2 vaccination	12		(20)	
Second SARS-CoV-2 vaccination	3		(5)	

Data analyses were performed using IBM SPSS Statistics (Version 27) software [19].

Clinical and sociodemographic data as well as number of symptomatic patients over the follow-ups together with the type of the symptoms and their severity were analyzed descriptively and expressed as percent, mean, standard deviation (SD) or median (MD) with minimum and maximum. Effect on daily activities, economic impact and mental health were also analyzed descriptively. Because some of the variables were assessed categorically, the mode was reported in addition to the MD. To determine associations between the presence of symptoms and the development of PC as categorical variables, we performed Chi-square tests for contingency tables. Accordingly, we compared the observed frequencies with the expected frequencies in case of independence. To quantify the strength of the associations following a significant chi-square we determined Cramer ‘s V. The significance level was defined as *p*-value < 0.05.

### Definition of Post-Covid

For further data analysis, we define PC analogously to the WHO preliminary case definition. According to this, PC is defined as symptoms that persist for usually three months after a SARS-CoV-2 infection (Fig. 1: Red Area), or symptoms that appeared after an acute SARS-CoV-2 infection, persist for another two months, and cannot be explained otherwise [6]. The number respectively portion of participants still suffering from at least one symptom according to this definition was our primary outcome measure.

## Results

### Study population

During the recruitment period, approximately 1980 individuals in Wuerzburg and surroundings were tested positive (information provided by the health department); all individuals received an invitation to the study from the local health department. A total of 60 individuals were included in the study (response rate 3.0%). The study population included 24 (40.0%) male and 36 (60.0%) female patients with a median age of 43 years (Table 1).

### Prevalence of COVID-19 typical symptoms

Over time, the number of symptomatic patients decreased to 78.3% at week 4 (Long-COVID threshold), while 48.3% still reported COVID-19-related symptoms at week 12 (PC threshold) [6]. At week 24, 40.0% of patients still complained about symptoms.

**Fig. 2:**
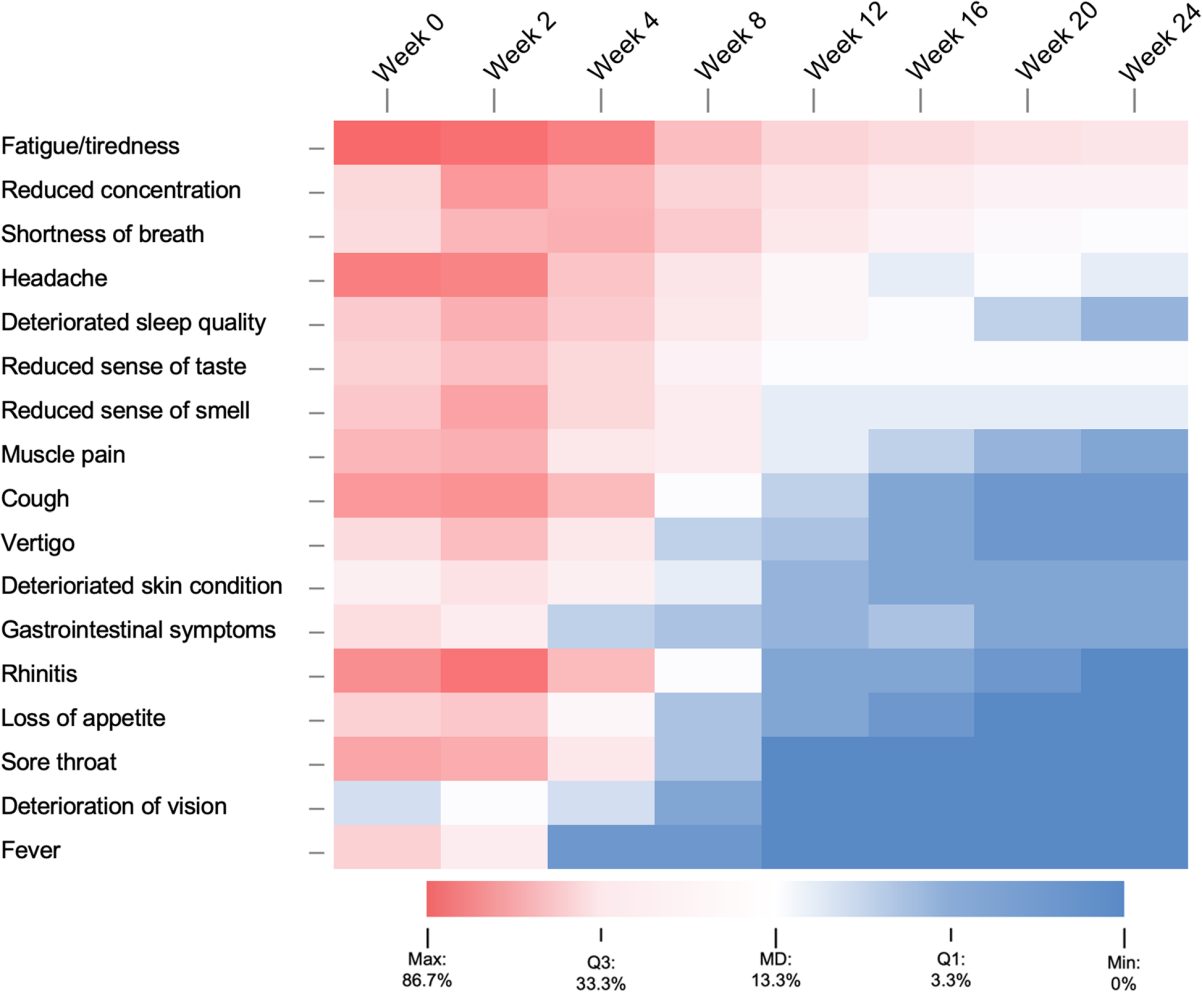
Symptom frequency during the survey period. High frequency is marked in red, low in blue. The variables are sorted in descending order according to their frequency in week 12 (threshold for PC as defined by WHO) respectively according to their influence on PC from very high influence to very low influence. Percentages refer to the proportion of patients from the total

Most prevalent symptoms reported at week 12 were fatigue/tiredness (33.3%) reduced concentration (26.7%) and shortness of breath (23.3%). In addition, 71.6% of patients reported limited mental and physical performance resources at the start of the study, which persisted in 25.0% until week 12 and in 13.3% until week 24. The relative frequencies of individual symptoms at the respective time points are shown in Fig. 2.

### Severity

The most frequent symptoms were not simultaneously those with the strongest intensity. At week 12, reduced sense of smell, fatigue/tiredness, loss of appetite, reduced sense of taste, and rhinitis were the symptoms with the strongest expression. In general, symptoms were rather mild at this time point, 72.7% of patients had very mild to mild symptoms, and only 27.3% had moderate to very severe symptoms.

### Predictive factors for post covid

**Fig. 3:**
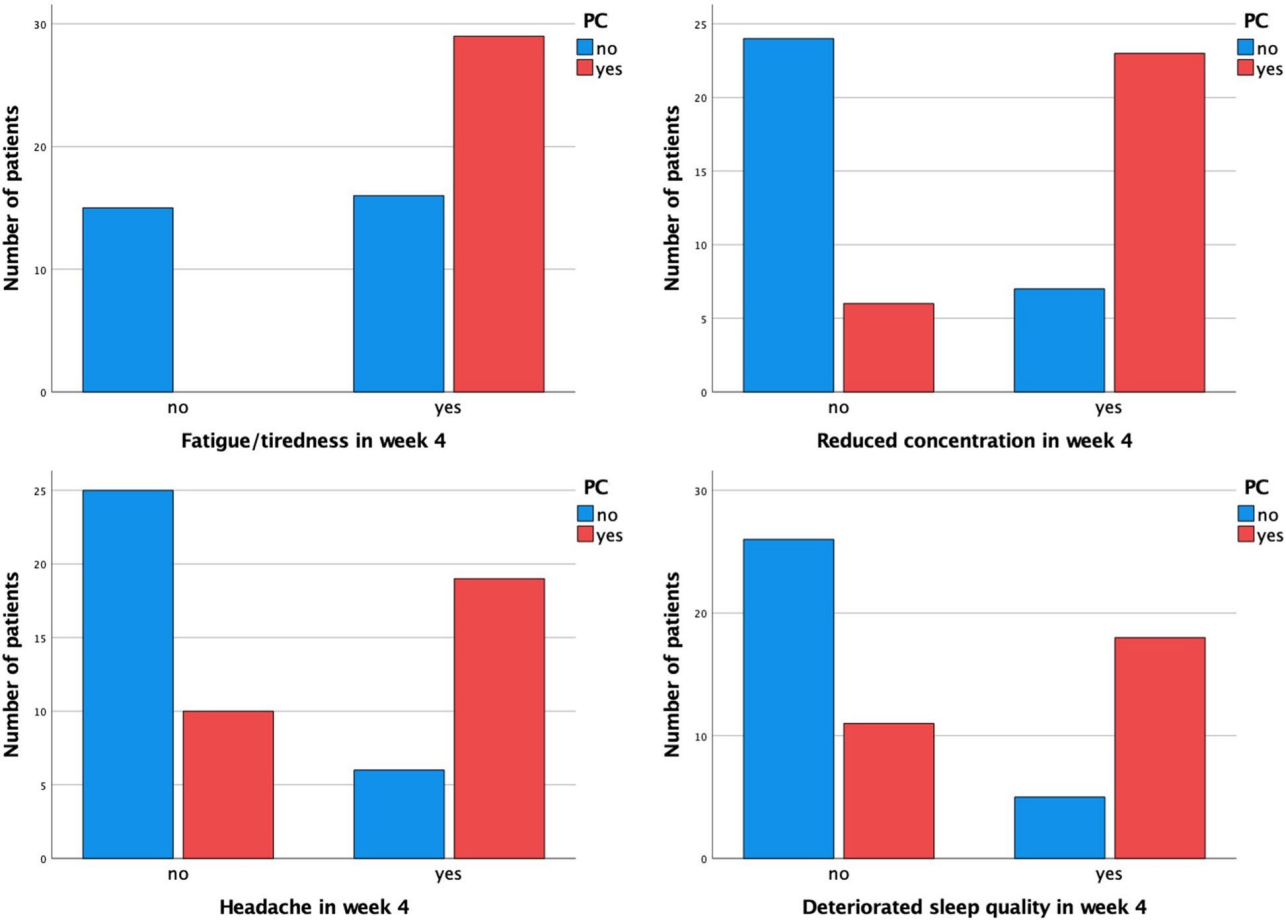
Occurence of PC (yes/no; red/blue) in dependence of the presence of the four significant predictive symptoms as assessed in week 4. (X-axis; yes/no)

In week four, we checked whether the predominant symptoms had predictive significance for the later occurrence of PC. As you can see in Table 2, in particular, the symptoms fatigue/tiredness, reduced concentration, headache and deteriorated sleep quality showed a high significance. The determined Cramer V attested a moderate to strong (0.47–0.57) association for these four symptoms. A visualization of the chi-square tests for the four significant symptoms can be found in Fig. 3. In addition to the symptoms, we also analyzed the correlation between PC and sociodemographic factors such as age, gender and comorbidities. However, no significant correlations were found.

**Table 2:** Significances of the different symptoms at week 4 in relation to a possible PC according to the respective chi-square tests for contingency tables. The variables are sorted in ascending order according to their *p*-value. Values rounded to second decimal place. *P-value after alpha adjustment using Bonferroni-Holm correction

Symptoms	*p*-value	*p*-value α-adj.*	Cramer V
Fatigue/tiredness	< .005	< .005	.56
Reduced concentration	< .005	< .005	.57
Headache	< .005	< .005	.47
Deteriorated sleep quality	< .005	< .005	.47
Reduced sense of taste	.01	.09	.35
Reduced sense of smell	.01	.08	.35
Deterioriated skin condition	.01	.08	.35
Cough	.02	.21	.30
Loss of appetite	.03	.25	.28
Shortness of breath	.04	.30	.27
Muscle pain	.05	.34	.26
Deterioration of vision	.07	.43	.23
Sore throat	.17	.87	.18
Rhinitis	.20	.80	.17
Fever	.30	.89	.14
Gastrointestinal symptoms	.59	1	.07
Vertigo	.89	.89	.02

### Effect on daily activities and economic impact

**Fig. 4:**
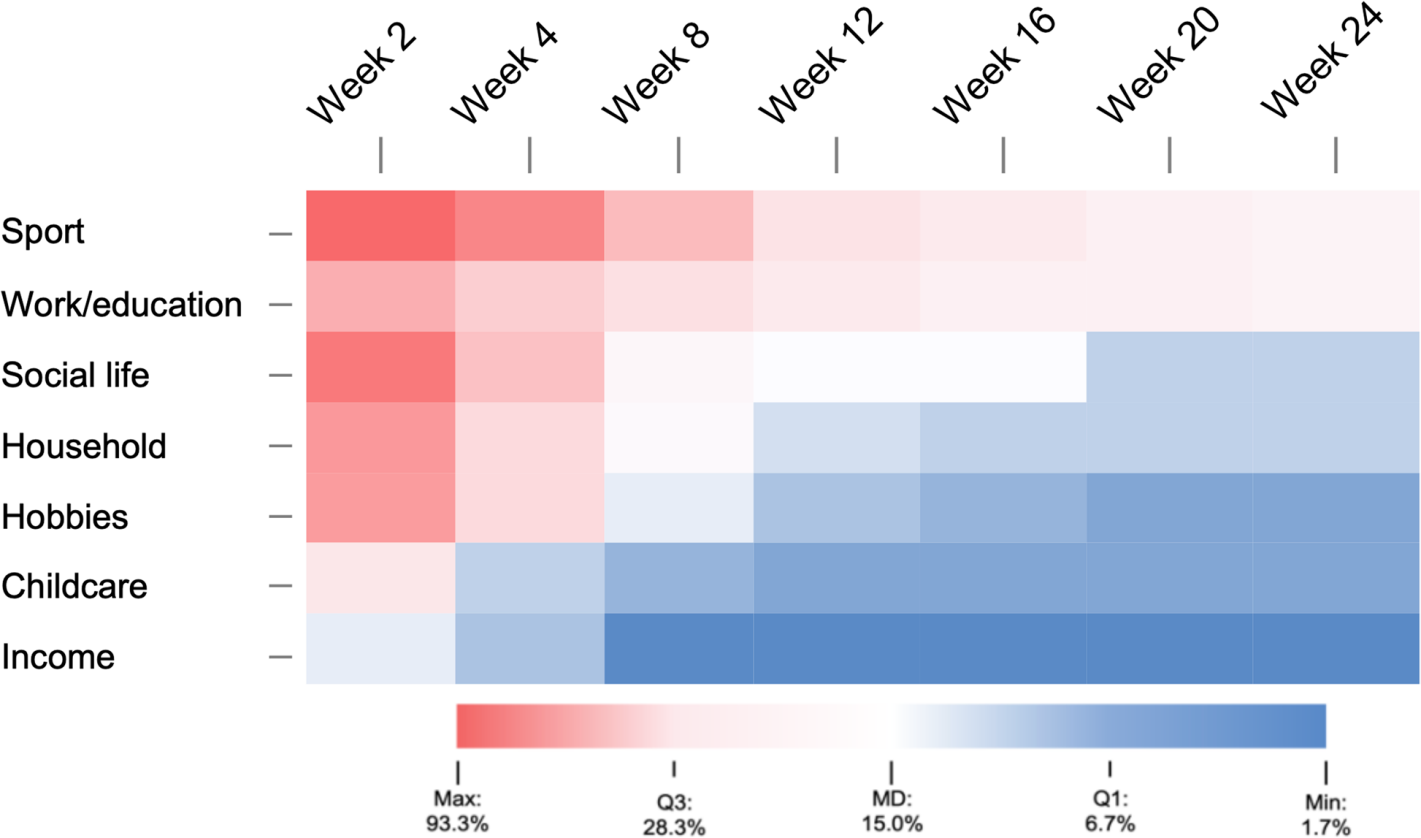
Percentage of patients reporting restrictions on various daily activities during the survey period. (regardless of their intensity, closed question). High number of restrictions are marked in red, low in blue. The variables are sorted in descending order according to their effect in week 12

At the beginning, most frequent limitations in daily life were reported in the categories of sports (93.3% at week 2) and social life (85.0% at week 2), while after six months sports (20.0%) and work (20.0%) were primarily negatively affected and in total 21.7% of patients had to reduce activities at home or at work due to symptoms of PC (see Fig. 4).

Frequently, symptomatic COVID-19 was associated with an increased economic burden for the employee as well as the employer and the health insurance. Over a quarter of participants (28.0%) reported a temporary inability to work beyond the official quarantine period (at that time two weeks). In such cases, patients were usually unable to perform their professional work for MD 11 to 15 working days (Min < = 5, Max > 20; Mo. 6–10) in addition to the official quarantine period.

Furthermore, an increased use of outpatient medical consultations was observed. More than two thirds (70.0%, *n* = 42) of the study participants visited a general practitioner or other specialist during the follow-up period due to COVID-19.

### Impact on Mental Health

**Fig. 5:**
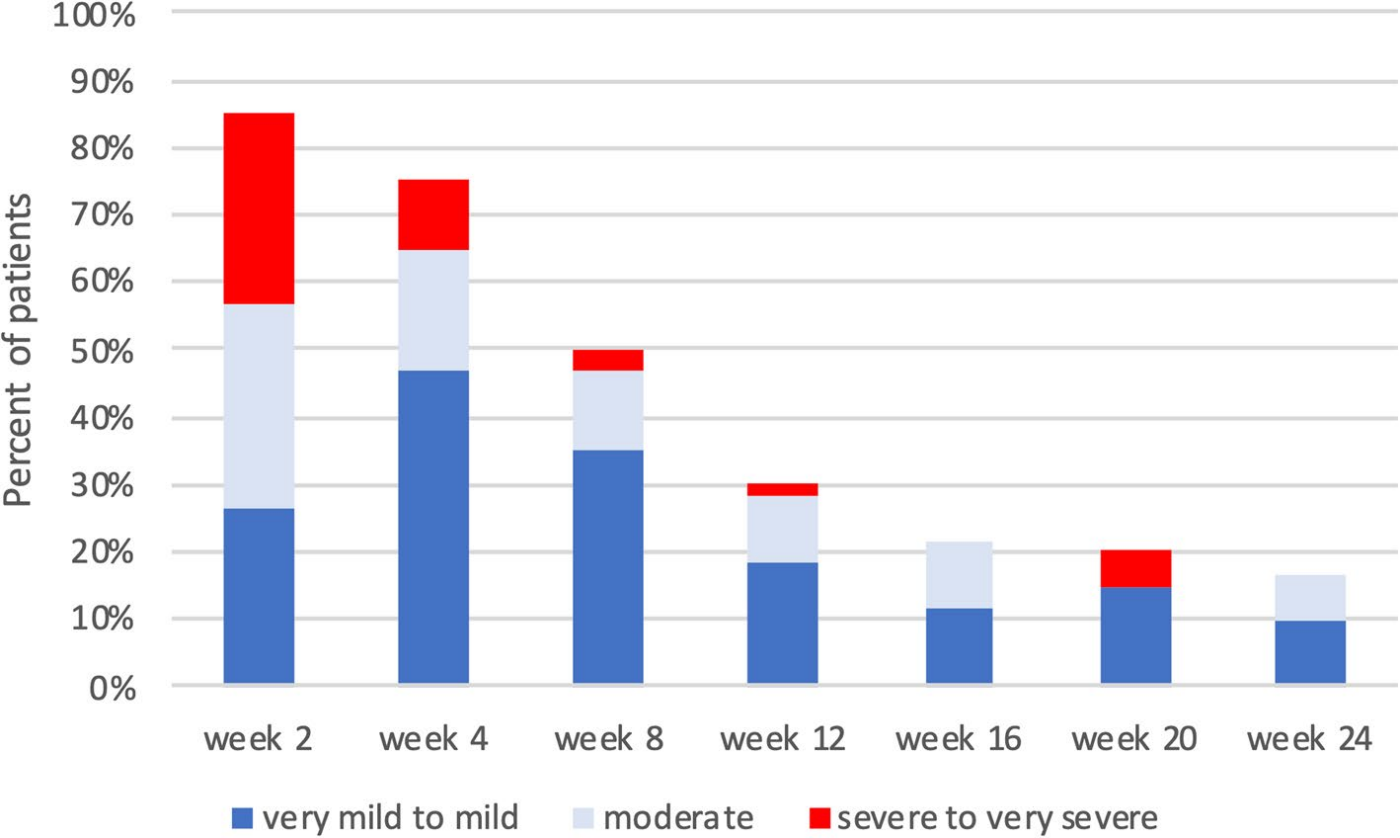
Course of the number of affected persons and the respective intensity of the general psychological symptoms during the survey period

The proportion of patients with mental symptoms such as anxiety, restlessness or depressive mood was 73.3% at the beginning of the study, increased to 85.0% by week 2 and then gradually decreased to 30.0% at week 12, and 16.7% after 24 weeks. However, these figures only relate to people who were still included in the follow-up (at week 24: 24/60). People who were no longer followed up because they were previously symptom-free were no longer surveyed. The distribution of intensities over the study period are shown in Fig. 5.

After six months, at the final interview, all 60 test subjects were interviewed, including those who had previously dropped out because they were symptom-free. Here, still 21.6% reported anxiety and 11.6% subjective depressive symptoms due to COVID-19. Furthermore, 40.0% were concerned that their health will deteriorate again or not completely recover because of COVID-19. As a validated instrument, the GAD-7 questionnaire was applied, which attested a minimal or mild anxiety symptomatology for 90.0% and moderate or severe anxiety symptomatology for 10.0% [18].

## Discussion

This prospective survey aimed to investigate the extent of symptoms and daily restrictions in the course of COVID-19 in 60 non-hospitalized patient whereas we have put a major focus on mental symptoms and the effects on daily life and society.

Accordingly, 48.3% of the participants developed PC (WHO definition) [6]. It appeared that PC, which is purely defined by persisting physical symptoms, cannot be limited to these disorders, additionally it directly or indirectly affects the quality of life of the individual. After six months 20.0% still reported restrictions in everyday activities such as sports, education or work. In addition 21.6% continued to suffer from anxiety and more than 40.0% continued to be concerned about their health.

### Post-COVID prevalence in outpatient care

A single term for symptom persistence after acute SARS-CoV-2 infection has yet to be found. Currently, there are many different terms such as post-acute COVID, chronic COVID syndrome, or the more commonly used Long-COVID (LC) and PC [20, 21]. The definition criteria here are often different and inconsistent between studies. This makes it fundamentally difficult to put data from different studies together.

According to WHO; fatigue, shortness of breath or breathing difficulties, memory-, concentration-, or sleep-problems are the predominant PC symptoms [22]. This is largely consistent with our data. A systematic review of 37 included studies on PC reported that common long-lasting symptoms also include loss of smell, taste, and cough, which tended to play a minor role in our cohort [23]. The causes may be different SARS-CoV-2 variants or the vaccination status of the infected persons. It should also be added that the meta-analysis cited showed that 3% to 74% of patients reported persistent smell and taste disturbances. The wide range here also indicates that the evidence regarding these symptoms is very variable [23]. The PC prevalence reported in the literature shows an enormous range, which can be explained by methodological differences as well as differences in patient demographics and comorbidities [11]. Standardized syndrome and symptom definitions as well as operationalizations are needed for more accurate data in the future. Another factor that may lead to differences in prevalence is the viral variant. Studies have shown that LC occurs less frequent in the Omicron variant than in the Alpha variant (21% vs. 50.5%) with the latter being predominant in our study population [24, 25].

### Predictive factors for PC

Several risk factors for PC and LC have already been described. These include, for example, female gender, obesity, smoking and a wide range of comorbidities [14, 26, 27]. However, little has been reported on early symptoms that have an effect on the long-term course of the disease, although this information may be useful for primary care physicians to provide early preventive or counseling measures. We were able to show that the presence of fatigue/tiredness, reduced concentration, headache and deteriorated sleep quality at week 4 is predictive for later PC. This finding is not very surprising, since these symptoms are those that are predominantly still present at week 12 or persist until then, whereas other symptoms show a significant decrease between weeks 4 and 12 (see Fig. 2). The fact that persistent fatigue/tiredness and/or reduced concentration are predominant symptoms on PC should give reason to further characterize the neurobiological background in order to create adequate treatment strategies [10].

### Effect on daily activities

Relevant influences on daily activities such as negative influence on sports, education or work, which were present in 20.0% in our group, were confirmed in a study by Jacobson et al. in 2021 on functional impairment, which showed health-related disabilities in work (35%) and general activities (46%) of the non-hospitalized patients after 3 to 4 months [28]. One reason for the significantly higher prevalence in this study compared to ours could be a different SARS-CoV variant. In addition, the WPAI (Work Productivity and Activity Impairment Questionnaire) questionnaire used by Jacobson et al. has a different question structure [29]. However, it seems likely that restrictions in daily life, together with physical symptoms, have a negative impact on mental health.

### Underreported mental symptoms

A meta-analysis showed that the most common mental symptoms after COVID-19 are anxiety, sleep disorders and depression [30]. This can be confirmed partly by this study. We showed that six months after infection, 21.6% still suffered from mental impairments such as anxiety, while up to 40.0% were concerned that their health would deteriorate again or not completely recover. However, not only the patients themselves but also the doctors showed an increase in mental health problems. For example, an increase in anxiety and sleep disorders was reported among GPs during the first wave due to great uncertainty and physical and psychological stress [31].

Many studies do not survey mental disorders by using common diagnostic criteria. We decided to use the GAD 7 as a reliable test in the final survey and were able to show that 10.0% of the subjects suffered from moderate or severe anxiety symptoms. In a meta-analysis from 2021, Schou et al. were able to detect the severity of the disease, the duration of the symptoms, and the female gender as risk factors for a mental disorder. Fortunately, in the same study, as well as in our study, it could be shown that there is a tendency for symptoms to improve over time [32].

As an explanatory model for the development of PC the biopsychosocial model is discussed [33]. Here, biological factors (e.g., pre-existing conditions), health behaviors, psychological factors (e.g., social isolation, perceived threat), and social resp. situational factors (economic situation, availability of medical care) interact to define individual exposure and vulnerability. This determines the clinical and psychosocial (anxiety, depression, sleep disturbances) course of the disease which in turn influences the long-term physical and psychological consequences. It shows that COVID-19 and a resulting PC Syndrome is not a purely physical condition, but is based on a multifactorial disease development in which, in addition to physical aspects of the disease, social factors such as limited daily activities, isolation, and economic threat play a significant role [33, 34]. Even if a precise evaluation of vulnerability and environmental factors is useful and should be included in treatment, further research is needed to break down the pathophysiology of PC. On the one hand, to facilitate diagnosis (e.g. using biomarkers), to expand treatment options and, finally, to use laboratory-based diagnoses to counteract the stigmatization of PC patients, whose symptoms are often not taken serious [8, 35, 36].

### Impact on the economy and health care system

We were able to show that in particular the long duration of illness in PC presumably represents a financial burden for the German social systems. The prolonged illness leads to a longer incapacity to work (MD 11–15 days). During sick leave, employers in Germany had to pay full salary for a maximum of six weeks; if the duration of illness exceeds six weeks, the health insurances continue to pay the salary at a reduced rate of approximately 70% [37]. A representative data collection of a large German health insurance showed that PC patients with a mild course were on sick leave for an average of 90 days per year [38]. In comparison, the average sick leave of the insured was only 15 days [38]. In addition, it can be assumed that even after returning to work, the performance capacity may still be significantly limited and in some cases an occupation may no longer be possible due to the persistent symptoms and thus, an occupational disability is imminent or at least an occupational retraining is necessary. An economic burden is also confirmed by a German study. It estimates a production loss of 3.4 billion euros for 2021 in Germany. The loss in gross value added was calculated at 5.7 billion euros. Finally, it is estimated that in the medium term, 0.4 percent of employees will leave the labor market in whole or in part due to long/post-COVID [39].

### Strengths and limitations

In this prospective data collection, we were able to generate an almost gapless data set with the help of structured questionnaires, as well as personal regular long-term follow-up. Possible bias of the collected data due to different interviewers was excluded, as the standardized interviews were conducted exclusively by one person. Recruitment was conducted through the public health department, and every last individual who tested positive for SARS-CoV-2 during the recruitment period were invited to participate in this study without any exceptions. However, the need for active contact by patients does not eliminate the possibility of bias, as patients who actively choose to participate in a COVID-19 study may be more engaged with their disease than other patients.

Recruitment occurred when the alpha variant (B.1.1.7) was predominantly active with portions of around 90% in Germany [16, 17, 40]. Recent studies show that the Alpha variant (in contrast to the subsequent Omicron variant) is associated with more severe courses, prolonged symptoms and leads to both more LC and PC cases [24, 25, 41, 42]. In addition, our cohort was predominantly unvaccinated (80% unvaccinated). In vaccinated collectives, the expression of LC is significantly lower [43]. An initial dose of vaccine decreases the risk of LC by 13%, a second vaccination lowers it by an extra of 9% [43]. Thus, one must assume that collectives currently affected by COVID-19 face less severe consequences than reported in our study. In turn, our population seems to be very appropriate for studying PC. Also, our data may be historically valuable and specifically suitable for comparison with newer variants and pandemics. In general, it would be informative to conduct a survey of a similar cohort presently to gain a deeper comprehension of the disease expression and personal coping mechanisms.

Furthermore, we had no control group and only a relatively small study population which potentially effects on the statistical power. Due to the short establishment period of the study, we refrained from recruiting a control group. It would certainly be worth repeating the study with a larger cohort and control group. Data on symptoms were based on subjective assessments and were not collected by rational parameters, e.g., respiratory rate or physical fitness performance tests. The final application of the GAD-7 at the time of six months does not allow a comparison to the situation before the infection or at the beginning of the disease.

## Conclusions

At the beginning of COVID-19 Pandemic, variability and duration of lingering symptoms was completely unknown. In our early cohort we could show, that after 12 weeks, 48.3% of outpatients reported not having fully recovered from their infection. Not only physical symptoms seem to play a decisive role, but also mental health and restrictions in activities of daily life. Overall PC is influenced by multiple factors and poorly understood in its complexity and impact on everyday life. As our work is only a small cohort and not suitable for generalization, we hope that our findings highlight the need for further research in larger, more diverse cohorts to better characterize the long-term effects, especially in outpatient care.

## Supplementary Information

Supplementary Material 1.

Supplementary Material 2.

## Data Availability

The datasets generated and analyzed during this study are not publicly available due to further evaluation but are available from the corresponding author on reasonable request.

## Abbreviations

CALIP: COVID-19 and lingering symptoms in primary care patients
COVID-19: Coronavirus disease 2019
GAD-7: Generalized anxiety disorder 7
LC: Long-COVID
PC: Post-COVID
WHO: World Health Organization
SARS-CoV-2: Severe acute respiratory syndrome coronavirus type 2
