# Supplementary material for: Lingering symptoms in non-hospitalized patients with COVID-19 – a prospective survey study of symptom expression and effects on mental health in Germany
Source: BMC Prim Care. 2025 Apr 2;26:94. doi: 10.1186/s12875-025-02784-3 (PMC11963417; doi:10.1186/s12875-025-02784-3)
Supplement: Supplementary file 1 — Supplementary Material 1. [file 12875_2025_2784_MOESM1_ESM.docx]

Lingering symptoms in non-hospitalized patients with COVID-19 – a prospective survey study of symptom expression and effects on mental health in Germany

**Questionnaire: Week 0**

**Inclusion criteria**

- Laboratory-confirmed positive test for SARS-CoV2 (date: ________________)
- Mild or moderate symptoms compatible with COVID-19
- Maximum of two weeks since positive SARS-CoV2 test
- 18 years or older
- Patient is able and agrees to consent to the follow up protocol
- • Does patient meet all inclusion criteria? Yes  No

**Exclusion criteria**

Patient underwent inpatient treatment for COVID-19 disease

- Does the patient fulfill an exclusion criterion? Yes  No

**Patient characteristics**

- Gender male  female  diverse
- Age _______ years
- Size _______ cm
- Weight _______ kg
- BMI _______ (kg/m^2^)
- Living alone? yes  n0

If not, how many people live in your household? _______
How many of them are children/young people under the age of 18?

- Profession

Medical activity

Social activity (e.g. educator, teacher)

Office work

Craft/industrial activity

Pupil/student

Parental leave/housewife/man, caring for relatives

Not working or retired

other

- Smoking behavior: yes  formerly  no
- Comorbidities: yes  no

Type of comorbidity

Chronic respiratory disease (e.g. asthma, COPD, CF)
 Diabetes mellitus
 Cardiovascular disease (e.g. CHD, PAD, hypertension, apoplexy)
 Chronic gastrointestinal diseases (e.g. Crohn's disease)
 Autoimmune disease (e.g. rheumatism, post-transplantation)
 Neurological disease (e.g. Parkinson's disease, epilepsy, seizures)
 Tumor/cancer disease (also Z.n.)
 Others? _____________________________________________________

- Permanent medication

Blood thinning
Antihypertensives
Blood fat reducer
PPI
DM medication
Psychotropic drugs,
neuroleptics
Painkillers
Other

Vaccination status regarding

Flu/influenza

pneumococcus

COVID

Blood group (if known):

A Known source of infection yes  no

**Initiale Symptome von COVID-19**

- Cough yes  no
- Shortness of breath yes  no
- Fever yes  no
- Sore throat yes  no
- Fatigue/tiredness yes  no
- Headache yes  no
- Loss of appetite yes  no
- Muscle pain yes  no
- Gastrointestinal complaints yes  no
  (e.g. diarrhea, nausea/vomitin)
- Loss or reduced sense of taste yes  no
- Loss or reduced sense of smell yes  no
- Mental symptoms ) yes  no
  (e.g. anxiety, restlessness, depressive mood
- Deteriorated skin condition yes  no
- Deteriorated sleep quality yes  no
- Impaired memory yes  no
- Deteriorated general condition yes  no
- Other symptoms/complaints yes  no

**Questionnaire Week 2**

Have you been hospitalized for COVID-19 within the last week?

Yes  No

If yes, why? _________________________________________.

**Please rate the severity of the following symptoms you have experienced within the last week:**

- Fever

No  very mild  mild  moderate  severe  very severe

< 37,5°C 37,5°C-38,0°C 38,1°C-38,5°C 38,6°C-39,0°C 39,1°C-39,9°C ≥ 40,0°C

- Cough

No  very mild  mild  moderate  severe  very severe

- Shortness of breath

No  very mild  mild  moderate  severe  very severe

- Sore throat

No  very mild  mild  moderate  severe  very severe

- Fatigue/tiredness

No  very mild  mild  moderate  severe  very severe

- Headache

No  very mild  mild  moderate  severe  very severe

- Loss of appetite

No  very mild  mild  moderate  severe  very severe

- Muscle pain

No  very mild  mild  moderate  severe  very severe

- Gastrointestinal symptoms

No  very mild  mild  moderate  severe  very severe

- Loss of taste

No  very mild  mild  moderate  severe  very severe

- Loss of smell

No  very mild  mild  moderate  severe  very severe

- Mental symptoms (e.g. anxiety, restlessness or depressive mood)

No  very mild  mild  moderate  severe  very severe

Emotional sensitivity

No  very mild  mild  moderate  severe  very severe

- Deteriorated skin condition

No  very mild  mild  moderate  severe  very severe

- Deteriorated sleep quality

No  very mild  mild  moderate  severe  very severe

- Impaired memory

No  very mild  mild  moderate  severe  very severe

- Deteriorated general condition

No  very mild  mild  moderate  severe  very severe

- Other symptoms/complains _________________________________________

**Please rate the negative impact of your COVID-19 disease on the following activities:**

- Education/work

Not at all  slightly moderate  quite strong extremely

- Income

Not at all  slightly moderate  quite strong extremely

- Caring for children/grandchildren

Not at all  slightly moderate  quite strong extremely

- Household

Not at all  slightly moderate  quite strong extremely

- Sport

Not at all  slightly moderate  quite strong extremely

- Social life

Not at all  slightly moderate  quite strong extremely

- Hobbys

Not at all  slightly moderate  quite strong extremely

- Other activities problems/restricitons (please specify)

Not at all  slightly moderate  quite strong extremely

Has your state of health normalized? (As before the COVID-19 disease)

yes  No

If no, please describe why your health has not returned to normal? ____ _______________________________________________________________

Have you had any acute respiratory infections in the last week?

yes  No

Did you have symptoms similar to those at the start of COVID-19?

yes  No

How severe were the symptoms compared to the original COVID-19 symptoms?

Less heavy  Identical  more severe

Have you contacted your family doctor within the last week due to COVID-19 symptoms?

yes  No

Please enter the number of times you have contacted your family doctor:___

What advice did you receive? ________________________________________________

Have you consulted another specialist for COVID-19 symptoms within the last week?

Yes  No

If yes, which one(s)?___________________________________________________________

Have you taken any medication for your COVID-19 symptoms in the last week?

Yes  No

If yes, which one? ____________________________________________________________

**Questionnaire Week 8 - 24**

Have you had any symptoms due to COVID-19 in the last month?

Yes  No

Has your state of health normalized? (As before the COVID-19 disease)

yes  No

If no, please describe why your health has not returned to normal? ____ ___________________________________________________________________________

Have you been hospitalized for COVID-19 within the last week?

Yes  No

If yes, why? _________________________________________.

**Please rate the severity of the following symptoms you have experienced within the last week:**

- Fever

No  very mild  mild  moderate  severe  very severe

< 37,5°C 37,5°C-38,0°C 38,1°C-38,5°C 38,6°C-39,0°C 39,1°C-39,9°C ≥ 40,0°C

- Cough

No  very mild  mild  moderate  severe  very severe

- Shortness of breath

No  very mild  mild  moderate  severe  very severe

- Sore throat

No  very mild  mild  moderate  severe  very severe

- Fatigue/tiredness

No  very mild  mild  moderate  severe  very severe

- Headache

No  very mild  mild  moderate  severe  very severe

- Loss of appetite

No  very mild  mild  moderate  severe  very severe

- Muscle pain

No  very mild  mild  moderate  severe  very severe

- Gastrointestinal symptoms

No  very mild  mild  moderate  severe  very severe

- Loss of taste

No  very mild  mild  moderate  severe  very severe

- Loss of smell

No  very mild  mild  moderate  severe  very severe

- Mental symptoms (e.g. anxiety, restlessness or depressive mood)

No  very mild  mild  moderate  severe  very severe

Emotional sensitivity

No  very mild  mild  moderate  severe  very severe

- Deteriorated skin condition

No  very mild  mild  moderate  severe  very severe

- Deteriorated sleep quality

No  very mild  mild  moderate  severe  very severe

- Impaired memory

No  very mild  mild  moderate  severe  very severe

- Deteriorated general condition

No  very mild  mild  moderate  severe  very severe

- Other symptoms/complains _________________________________________

**Please rate the negative impact of your COVID-19 disease on the following activities:**

- Education/work

Not at all  slightly moderate  quite strong extremely

- Income

Not at all  slightly moderate  quite strong extremely

- Caring for children/grandchildren

Not at all  slightly moderate  quite strong extremely

- Household

Not at all  slightly moderate  quite strong extremely

- Sport

Not at all  slightly moderate  quite strong extremely

- Social life

Not at all  slightly moderate  quite strong extremely

- Hobbys

Not at all  slightly moderate  quite strong extremely

- Other activities problems/restricitons (please specify)

Not at all  slightly moderate  quite strong extremely

Have you had any acute respiratory infections in the last week?

yes  No

Did you have symptoms similar to those at the start of COVID-19?

yes  No

How severe were the symptoms compared to the original COVID-19 symptoms?

Less heavy  Identical  more severe

Have you contacted your family doctor within the last week due to COVID-19 symptoms?

yes  No

Please enter the number of times you have contacted your family doctor:___

What advice did you receive? ________________________________________________

Have you consulted another specialist for COVID-19 symptoms within the last week?

Yes  No

If yes, which one(s)?___________________________________________________________

Have you taken any medication for your COVID-19 symptoms in the last week?

Yes  No

If yes, which one? ____________________________________________________________

**Final interview (Week 24)**

**Symptomes**

1. Are you currently still experiencing symptoms of COVID-19?

Yes  No

- 1. If yes, which ones? ____________________________
  2. If yes, how severe?

No  very mild  mild  moderate  severe  very severe

1. Have your symptoms of an existing disease become more severe after COVID-19?

Yes  No

- 1. If yes, which ones? ____________________________

1. Have you developed any other new disease after COVID-19? (e.g. high blood pressure, allergies, diabetes mellitus, heart attack, mental illness)

Yes  No

- 1. If yes, which ones? ____________________________

**Emotions/mood**

1. Are you worried that your state of health will deteriorate again or not fully normalize due to COVID-19?

Yes  No

1. Do you have states of anxiety due to COVID-19?

Yes  No

1. Do you have depressive moods due to COVID-19??

Yes  No

- **Generalized Anxiety Score – 7 (GAD-7)**

How often have you felt affected by the following complaints in the last 2 weeks?

Feelings of nervousness, anxiety or tension

Not at all  Several days  More than half the days Nearly every day

- Not able to control or stop worrying

Not at all  Several days  More than half the days Nearly every day

- Worrying too much about different things

Not at all  Several days  More than half the days Nearly every day

- Trouble relaxing

Not at all  Several days  More than half the days Nearly every day

- Beeing so restless that is it hard to sit still

Not at all  Several days  More than half the days Nearly every day

- Becoming easily annoyed or irritable

Not at all  Several days  More than half the days Nearly every day

- Feeling afraid as if something awful might happen

Not at all  Several days  More than half the days Nearly every day

**Dealing with everyday life**

1. Are there any activities at home or at work that you can no longer perform due to persistent symptoms of COVID-19?

yes  no

- 1. If yes, at home or at work?
  2. If yes, which ones? ________________________

1. Do you have to reduce activities at home or at work due to symptoms of COVID-19?

yes  no

- 1. If yes, at home or at work?
  2. If yes, which ones? ________________________

1. Have you had to take time off work due to symptoms of COVID-19?

yes  no

- 1. If yes, how many days? (Exact number/total: ____)

</= 5 6-10  11-15  16-20  >20

**Influence of the SARS-CoV-2 vaccination**

1. Have you received a SARS-CoV-2 vaccination after SARS-CoV-2 infection?

yes  (time interval to positive SARS-CoV-2 test:__________) no

- 1. If yes, did the SARS-CoV-2 vaccination improve the symptoms you experienced during the follow-up period?

yes  no

- 1. If yes, did the SARS-CoV-2 vaccination increase the symptoms you mentioned during the observation period?

yes  no

**Therapeutic measures**

1. Have you seen a doctor for symptoms of COVID-19 within the last four weeks? yes no
   1. If yey, which ones?

General practitioner  Specialist outpatient clinic

1. Have you taken medication due to symptoms of COVID-19 within the last four weeks?

yes no

- 1. If yes, which ones? _______________________

1. Have you undergone inpatient or outpatient rehabilitation due to COVID-19?

yes  no

- 1. If yes, which one?

inatient  outpatient

1. Have you been prescribed therapeutic treatments (e.g. physiotherapy, occupational therapy, psychotherapy) due to COVID-19?

yes  No

- 1. If yes which ones? _______________________

1. Have you sought non-medical help (e.g. participation in a self-help group) due to COVID-19?

yes  no

15.1 If yes which ones? _______________________
