# Supplementary material for: Lingering symptoms in non-hospitalized patients with COVID-19 – a prospective survey study of symptom expression and effects on mental health in Germany
Source: BMC Prim Care. 2025 Apr 2;26:94. doi: 10.1186/s12875-025-02784-3 (PMC11963417; doi:10.1186/s12875-025-02784-3)
Supplement: Supplementary file 2 — Supplementary Material 2. [file 12875_2025_2784_MOESM2_ESM.pdf]

Symptom severities

Symptom	Week 2	Week 4	Week 8	Week 12	Week 16	Week 20	Week 24	Average	
Reduced sense of smell	3,7	2,6	2,2	2,9	2,9	2,4	2,4	2,4	0 none
Fatigue/tiredness	3,1	2,6	2,5	2,7	2,8	2,9	2,8	2,2	1 very mild
Reduced sense of taste	3,8	2,6	2,1	2,5	2,3	2,1	2,1	2,2	2 mild
Loss of appetite	2,4	2,0	1,5	2,5	1,0			1,4	3 Moderate
Rhinitis	2,7	1,9	1,6	2,5	2,0	2,0		1,6	4 Severe
Reduced concentration	2,1	2,4	2,5	2,4	2,5	2,7	2,5	2,1	5 Very severe
Deterioriated skin condition	2,1	2,3	2,0	2,3	3,0	3,0	3,0	1,8	
Muscle pain	2,1	1,9	1,5	2,3	2,4	3,3	3,5	1,5	
Headache	2,4	2,2	2,0	2,2	2,7	3,5	3,3	1,7	
Shortness of breath	2,4	2,4	2,1	2,1	2,3	2,4	2,5	2,0	
Deterioriated sleep quality	2,5	2,2	2,1	2,1	2,4	2,0	2,3	1,8	
Gastrointestinal symptoms	2,3	2,6	2,8	2,0	2,5	2,5	3,5	1,6	
Cough	2,4	1,9	1,9	1,8	2,5	3,0	3,0	1,5	
Vertigo	2,1	1,6	1,6	1,8	1,5	2,0	2,0	1,4	
Deterioration of vision	1,9	1,5	2,0					1,3	
Sore throat	1,9	1,5	1,5					1,0	
Fever	2,4	1,0	1,0					0,9	
Mentale symptoms	2,9	2,2	2,0	2,0	2,2	2,2	2,1	1,9	
